# Effects of dietary glutamine and arginine supplementation on performance, intestinal morphology and ascites mortality in broiler chickens reared under cold environment

**DOI:** 10.5713/ajas.17.0150

**Published:** 2017-06-26

**Authors:** Rahim Abdulkarimi, Mohammad Hossein Shahir, Mohsen Daneshyar

**Affiliations:** 1 Department of Animal Science, Faculty of Agriculture, University of Zanjan, Zanjan 45371-38791, Iran; 2 Department of Animal Science, Faculty of Agriculture, Urmia University, Urmia 57561-51818, Iran

**Keywords:** Ascites, Arginine, Broiler Chickens, Glutamine, Intestinal Morphology

## Abstract

**Objective:**

An experiment was conducted to evaluate the effects of dietary glutamine (Gln) and arginine (Arg) supplementation on performance, intestinal morphology and ascites mortality in broilers.

**Methods:**

A total of 675 day old chicks were randomly allocated to 9 experimental groups in a 3×3 factorial arrangement based on a completely randomized design with 5 replicates of 15 chicks. Three levels of dietary Gln (0%, 0.5%, and 1%) and Arg (100%, 130%, and 160% of Ross recommendation) supplementation were used in ascites inducing condition (15°C ±1°C) from 7 to 42 days of age.

**Results:**

Dietary supplementation of Gln increased body weight gain during grower, finisher and total periods (p<0.05) and increased feed intake during total period. Ascites mortality was decreased by Gln supplementation (p<0.05). Gln supplementation increased the villus height (VH) and crypt depth (CD) in duodenum and jejunum (p<0.05). Arg supplementation decreased CD in duodenum and jejunum, and increased ileum villus width (VW) and also VH/CD ratio in duodenum and jejunum (p<0.05). Both Gln and Arg increased the goblet cell number (GCN) in duodenum whereas Gln supplementation decreased GCN in jejunum and ileum (p<0.05). The Gln×Arg interaction were observed for right ventricle (RV)/total ventricular (TV) ratio, VH, VW, CD, VH/CD.

**Conclusion:**

It was concluded that dietary 0.5% Gln alone or along with 130% Arg of Ross requirement, improve the intestinal morphology and performance and hence decrease the ascites mortality in broiler chickens with cold induced ascites.

## INTRODUCTION

Ascites or pulmonary hypertension syndrome (PHS) is one of the main causes of mortality in modern broilers. It is estimated that ascites incidence may be as high as 20% of total mortality in broilers [1]. Due to selection for rapid growth, broiler chickens have a high metabolic rate and hence higher oxygen requirements. However, they have a marginal capacity of lung and cardiovascular systems to supply their high oxygen demand. This results in impaired ability to regulate the blood oxygen under extreme conditions such as low ambient temperature or high altitude and leads to hypoxemia, increased cardiac output, pulmonary hypertension, right ventricular hypertrophy and finally ascites and death [2]. Among the organs of broilers, gastrointestinal tract (GIT) is a highly metabolic active organ that demands high oxygen supply. The total oxygen demand of gut is not known for broilers, but in pigs it consumes about 25% of the total oxygen, while it represents only 5% of total body weight [3]. The negative effects of heat stress, oxidative stress and hypoxic conditions has been well documented on gastrointestinal function and metabolism of monogastric animals [4,5]. Therefore it can be postulated that improvement of gut integrity and function can have beneficial effects on broiler health and performance in ascites inducing conditions.

Recent studies have shown that supplementation of certain amino acids can promote gut function, gastrointestinal integrity and subsequently growth performance of broilers [6]. Glutamine (Gln) and arginine (Arg) stimulate gut development and improve the overall efficiency of the chicken GIT [7,8]. The benefits of dietary Gln supplementation on performance, gut morphology and immune responses have recently been reported in poultry [7,9–11]. Although Gln is a non-essential amino acid, it is considered as an essential amino acid under challenging conditions [12]. Gln is the main energy source for rapidly proliferating cells such as intestinal enterocytes [13] and plays an important role in the intestinal immune system, mucin synthesis, maintenance of mucosal structure and integrity of epithelial barrier against pathogen attacks [11,14].

Arg is an essential amino acid for birds which cannot be synthesized by de novo routes, and birds are dependent on dietary supply [15]. Arg has been recognized as one of the functional amino acids that regulates key metabolic pathways and necessary for maintenance, growth, reproduction, and immunity [15]. Arg contributes in the synthesis of several crucial compounds such as nitric oxide (NO), creatine, polyamines, citrulline, ornithine, proline, glutamate, and enhances secretion of insulin, growth hormone and insulin-like growth factor 1 in animals [15,16]. Arg involvement in polyamines synthesis plays an important role in development of small intestinal mucosa and growth [17]. Arg supplementation, has improved the production performance and intestinal morphology of broilers [15]. Supplementation of 10 g/kg Arg, has reduced the susceptibility to PHS and improved gut function through increase in villus height (VH) of small intestine in broilers reared at high altitude [8].

The beneficial effects of Gln and Arg supplementation on production performance and gut function has been accepted under normal rearing conditions. However, not enough information is available regarding the effects of dietary supplementation by Gln and Arg, alone or in combination, under ascites inducing cold ambient conditions. Therefore, current study was conducted to assess the effects of different levels of these two amino acids on performance, intestinal morphology and ascites incidence in broilers reared under cold environment.

## MATERIAL AND METHODS

All of the experimental protocols were reviewed and approved by the animal care and use committee of the University of Zanjan, Iran. A total of 675 day old female chicks (Ross 308, 45±3 g) were obtained from a commercial hatchery and reared in an environmentally controlled house at the altitude of 1,670 meters under ascites inducing cold condition after 7 days until 42 days of age. At the beginning of experiment, birds were randomly allocated to 9 treatments with 5 replicate pens and 15 chicks for each based on a completely randomized design in a 3×3 factorial arrangement (glutamine×arginine levels). Three levels of dietary Gln (0%, 0.5%, and 1%), and Arg (100%, 130%, and 160% of Ross 308 recommendation) supplementation were used. A basal diet was formulated with corn-soybean meal according to Ross (308) recommendation (Table 1) and supplementation of Gln and Arg was performed on top of the basal diet. The L-Arg hydrochloride (99.6% purity) and L-Gln (98.7% purity) were purchased from Vitajoy company, China (B13-102, NO.192, Tinglan Lane, Suzhou, Jiangsu, China). Water and feed were provided *ad libitum* and birds were subjected to 23 h light and 1 h dark throughout the experimental period. The birds were raised under standard conditions during the first week of age, but in order to induce ascites, temperature was reduced to 26.0°C±1°C, 20.0°C±1°C, and 15.0°C ±1°C at 7, 14, and 21 d of age respectively and maintained at 15.0°C±1°C until the end of the experiment [2]. The average feed intake (FI, g/bird/d), body weight gain (BWG, g/bird/d) and feed conversion ratio (FCR) were determined during the starter (1 to 10 d), grower (11 to 24 d), finisher (25 to 42 d), and whole (1 to 42 d) periods.

During the experiment, mortality was recorded daily and all of the dead chickens were necropsied for ascites diagnosis based on amber colored fluid accumulation in abdominal cavity and pericardium, vascular congestion and ratio of right to total ventricular (TV) weight more than 0.29 [18]. The dead birds without the above mentioned symptoms were categorized as non-ascites mortality. Daily ascites mortality was recorded for each pen separately and at the end of the experimental period ascites mortality percentage calculated by number of dead birds due to ascites/total birds in each pen [19]. At 42 days of age, two birds per pen were selected, weighed and killed by decapitation to obtain the visceral organs weight (liver, gizzard, pancreas, and spleen), heart and intestinal segments. The liver, gizzard, pancreas and spleen removed, weighted and proportional weights of each organ were calculated relative to live weight. The heart was dissected and proportional weight of TV and right ventricle (RV) was determined RV to TV ratio was calculated. The small intestine was removed (from gizzard to ileocecal junction) and emptied of digesta contents. Then, the length and weight of whole intestine were measured. For experimental samples, small intestine divided into 3 segments and two cm of the middle portions of duodenum (from gizzard to entry of the bile and pancreatic ducts), jejunum (from entry of the ducts to diverticulum vitellinum), and ileum (from diverticulum vitellinum to the ileocecal junction) were cut, digesta was carefully washed away using normal saline, and samples were placed in 10% buffered formalin. Samples were dehydrated by alcohol, infiltrated with xylene and then embedded in paraffin. The paraffin sections were cut in 7.00 μm size by microtome and then stained by hematoxylin and eosin for VH, villus width (VW), and crypt depth (CD) measurement. The goblet cells numbers (GCN) were identified by periodic acid-schiff stain [20]. At the end, these samples were examined by multiple magnification (optical lens 400× and 1,000×) and morphometric analysis of digital photos of light microscopy were performed by means of image J analysis software [20]. The VH was measured from the top of the villus to the top of the lamina propria. The VW was averaged from VW at one-third and two-third of each villus. The CD was measured as the distance between the base of the villus to the sub mucosa [8]. After measuring of VH and CD, the VH to CD ratio (VH/CD) was calculated separately for each part. All procedures including the uses of birds, management and care were in compliance with the European parliament and the European Council Directive regulations on the protection of animals used for scientific purposes (2010/63/EU).

The data were analyzed by the general linear model procedure of SAS (SAS Institute, 2003, Version 9.1.2) and difference between treatments means were evaluated by Tukey’s test at a significance level of 5%. The following model was used:

$${\text{Y}}_{\text{ijkl}}=\mu +{\text{A}}_{\text{i}}+{\text{G}}_{\text{j}}+{\text{AG}}_{\text{ij}}+{\text{e}}_{\text{ijkl}}$$

In which A_i_ (Arg level) and G_j_ (Glu level) were considered as the main effects and AG_ij_ as the interaction.

## RESULTS

### Performance and ascites mortality

The effects of dietary Arg and Gln supplementation on performance, ascites mortality and RV/TV are presented in Table 2 and 3. There were no significant differences between treatments in FI during the experimental periods but the main effect of Gln supplementation was significant during the total period (p<0.05) and the birds fed the medium level of Gln (0.5%) had higher FI. Dietary supplementation of 0.5% Gln had significant effect on BWG during the grower, finisher and total periods (p<0.05). The birds fed 0.5% Gln+160% Arg in diet had the highest BWG during grower, finisher and total periods (p<0.05). No interactions were found between the Gln and Arg on FI, BWG, and FCR. Supplementation of 0.5% Gln significantly reduced the FCR during finisher period (p<0.05). The experimental protocol was successful in ascites induction (ascites mortality of 46.6% in the control group). Ascites mortality reduced by 0.5% Gln supplementation (p<0.05) but not by Arg supplementation. The lowest ascites mortality (14.67%) attributed to 0.5% Gln+130% Arg experimental group (p<0.05).

### Small intestinal segments and visceral organs

The effects of dietary Gln and Arg supplementation on small intestinal length, small intestinal weight and visceral organs weight are summarized in Table 4. Dietary Arg and Gln supplementation had no significant effects on the length and proportional weight of small intestine and on the visceral organs proportional weights. Furthermore, there was no significant interaction between Arg and Gln levels for the above mentioned parameters.

### Small intestinal morphology

Effects of dietary Gln and Arg supplementation on the morphology of small intestinal segments are presented in Table 5. Dietary Gln affected the VH in duodenum and jejunum (p< 0.05). The birds fed the 0.5% Gln along with 130% Arg had the highest VH as compared to the birds in other treatments. Increasing the Gln level to 0.5% increased the VH only at the 130% Arg level whereas this increase was not observed at the other levels of Arg (Gln×Arg interaction). The VH of ileum was not affected by Gln or Arg supplementation. No effects of Arg and Gln or their interaction were found on VW in the duodenum and jejunum. VW of ileum increased by Arg (p< 0.05) and this effect was observed only at 0.0% and 0.5% of Gln (Gln×Arg interaction). Gln supplementation changed the CD in duodenum and jejunum (p<0.05). The 0.5% Gln+130% Arg fed birds had the highest CD in both the duodenum and jejunum (Gln×Arg interaction, p<0.05). No effects of Arg and Gln or their interaction was detected for CD in ileum. The VH/CD ratio was increased by Arg supplementation in duodenum and jejunum (p<0.05). At the medium Gln level (0.5%), increasing the Arg level to 160% increased the VH/CD ratio (Gln×Arg interaction, p<0.05). No effect of Arg, Gln and their interaction was observed on VH/CD ratio in ileum. Both the Gln and Arg affected the goblet cell number (GCN) in duodenum (p<0.05) and the 0.5% Gln+130% Arg and 1.0% Gln+ 160% Arg fed birds had higher GCN. Only Gln affected the GCN in the jejunum and ileum (p<0.05). No interaction between Arg and Gln was observed for GCN in all intestine parts (p>0.05).

## DISCUSSION

The beneficial effects of Gln supplementation on BWG and intestinal morphology in the recent experiment have been shown. This results supported by Bartell and Batal [11] and Soltan [9], who observed that consumption of 1.0% Gln in broilers, resulted in longer villi height and higher relative weights of duodenum and jejunum, and consequently higher BWG. In stressful conditions with high ambient temperature, Jazideh et al [7] reported that dietary supplementation of 0.5% Gln, caused a longer duodenum and jejunum VH and higher BWG. Nassiri Moghaddam and Alizadeh Ghamsari [21] observed that 1.0% and 1.5% dietary Gln supplementation increased the duodenum and jejunum VH, VW, and villus surface area of broiler chickens at days 21 and 42 of age. It has been well documented that maximum development of the small intestine occurs at 10 first day of age [22], therefore adequate feeding in the first days has an important role in broiler chicken performance [10]. Gln is an important substrate or energy source for maturation of fast proliferating cells such as enterocytes and hence its supplementation in the first days of age may have activated the cell mitosis and caused the higher VH. It has been stated that the longer VH and villous surface area positively related to BWG and growth performance [23, 24]. Hence the better performance of 0.5% Gln fed birds in current experiment is due to the higher villus length. The large surface area by higher VH results in better fed utilization [11] and consequently higher nutrient absorption that reflected in higher BWG and feed efficiency.

The Gln fed birds had the deeper CD in current study. Our results are inconsistent with those of Murakami et al [10] and Soltan [9] who reported that CD was shorter with Gln consumption. However, Xaio et al [4] stated that 1.0% Gln significantly increased the VH, VW, villus surface area, and VD. The higher CD of Gln consuming birds in our experiment may be associated with increased VH [4].

In a consistent with performance and intestine morphology, ascites mortality was affected by dietary Gln and the Gln fed birds had the lowest mortality. Nascimento et al [25] indicated that increasing the glutamine consumption (0, 5, 10, 15, and 20 g/kg) linearly reduced the coccidiosis incidence from days 1 to 21 of age. Consistently, Fathi et al [26] indicated a decreased total ascites mortality (38% vs 25%) with the consumption of 100 g/kg Gln. Similarly, the reduction of ascites incidence in Gln fed birds in the current study might be associated with improved O_2_ utilization of gut. Moreover, Gln supplementation might have improved the intestinal lumen health, increased the intestinal integrity against pathogens, improved immune responses with secretion of IgA and had protective effects on jejunum against oxidative stress as indicated by morphological traits [4,11]. It has been estimated that almost 20% of the total energy expenditure by animal is related to intestinal epithelium maintenance [27]. Therefore, the body net energy and oxygen avialability may be enhanced while epithelial turnover or intestinal lumen damage decreased. These results suggest that Gln may reduce O2 utilization by gut and allowing birds to use more oxygen and maintain their current high metabolism in cold temperature. In supporting this discussion, Santose et al [5] reported reduced ascites incidence in prebiotic treated birds under hypoxic condition (high altitude) and suggested that prebiotic treatment may revoke the effect of hypoxia in broilers along with stimulation of intestinal development and availability of more oxygen for body metabolism.

Although Arg supplementation did not affect the performance and ascites mortality in the current experiment but ileal VW, duodenum, and jejunum VH/CD ratio was increased and jejunum CD decreased by dietary Arg addition. Saki et al [28] reported no significant effect of 1.5 g/kg Arg on performance of broilers in hypobaric condition. Moreover, Khajali et al [8] indicated that dietary consumption of 10 g/kg Arg did not change the broiler performance at hypoxic condition (high altitude). In agreement with current results, Murakami et al [15], showed that dietary Arg (10%, 20%, 30%, and 40% higher than NRC recommendation) during starter period, improved the intestine mucosa development and even production performance. Xaio et al [4] indicated that 1% Arg supplementation exhibited significantly higher ratio of VH to CD and total antioxidant capacity activity and better morphological structure of rat jejunum under oxidative stress. Arg involvement in polyamines (putrescine, spermine and spermidine) synthesis which is known as biogenic amines plays an important role in development of small intestine, colonic mucosa, cell division and tissue growth [17]. Khajali et al [8] reported that dietary Arg supplementation (10 g/kg) increased blood NO, duodenum and jejunum VH, VW and surface area, and decreased RV/TV ratio and consequently reduced PHS in broiler under hypoxia condition. Higher VH and VH/CD ratio may be related with polyamines production and activation of cell mitosis or NO by additional levels of Arg supplementation which enhanced the gut development and nutrient absorption in small intestine.

In current experiment, both the Gln and Arg caused the greater GCN of the intestine especially duodenum. Goblet cells synthesize and secrete mucin and play crucial role in integrity and protection of epithelium [29]. Mucus generated by GCN is the first defense line against intestinal pathogens [29]. Duodenum is the first segment in small intestine with the faster cell renewal which receives the physical and chemical substrate after stomach. Although the main reason for the higher GCN in duodenum by Gln And Arg is not known yet, stimulation of this segment by supplementation of both the amino acids possibly caused the higher mucus secretion or an increase in goblet cell turnover.

No information is available regarding the intestinal GCN under ascites condition especially in amino acids supplemented diets. In a stressful condition under Salmonella Typhimurium challenge, higher goblet cell density and lower villus surface area have been observed in broilers [30]. Gln is an important anti stress amino acid [12] that nourishes the intestinal enterocytes and immune cells [13]. The lower GNC in jejunum and ileum of Gln fed birds in current experiment can be related to the beneficial effects of this amino acid on immunity regulation of small intestine segments which may be provided specific condition in intestinal lumen and have decreased the mucin secretion.

Although many beneficial effects of Gln (0.5%) and Arg (130%) supplementation alone or together were observed for many parameters (performance, intestine morphology, and ascites mortality) in current experiment, these results were not supported by an additional level of Gln (1%) or Arg (160%). This phenomenon may be related to the possible negative effects of higher levels of Gln and Arg on broilers. It has been shown that the 1.5% and 2% Gln [9] or 4% Gln [11] had toxic effects and resulted in body weight depression in broilers. Gln acts as a precursor for ammonia synthesis in the gut and kidney and is a nitrogen shuttle which protects the body from high levels of ammonia [31]. In birds, ammonia is excreted in urine in the form of uric acid and Gln is involved in uric acid synthesis [32]. A possible explanation for the lower ascites mortality of 0.5% Gln+130% Arg fed birds than other combinations of Gln and Arg, may be related to the lower excreted nitrogen which may hypothetically cause the reduced oxygen need for extra nitrogen metabolism.

## CONCLUSION

According to the results of the current experiment, the supplementation of diet with 0.5% Gln alone or in combination with Arg supplementation at 130% of Ross recommendation increased the villus length and decreased the muscular layer of intestine, improved the performance and consequently decreased the ascites mortality in broiler chickens with cold induced ascites. Higher levels of Gln (1.0%) and Arg (160%) alone or in combination, does not positively affect the ascites mortality.

## Figures and Tables

**Table 1:** Ingredient and chemical composition of basal diets

Items	Starter (1–10 d)	Grower (11–24 d)	Finisher (25–42 d)
Ingredients (%)			
Corn (7.34%)	44.029	52.05	58.445
Corn gluten meal (52.19%)	9.409	0.0	0.0
Soybean meal (45.45%)	39.369	40.078	34.319
Soy oil	2.392	3.815	3.397
Dicalcium phosphate	2.223	1.956	1.811
Calcium carbonate	1.219	0.969	0.955
L-lysine HCl (78%)	0.242	0.029	0.02
DL-methionine (99%)	0.214	0.245	0.207
Vitamin and mineral premix1)	0.5	0.5	0.5
Salt	0.255	0.326	0.326
Sodium bicarbonate	0.149	0.031	0.02
Total	100	100	100
Calculated analysis			
Dry matter (%)	89.801	89.535	89.17
Metabolizable energy (kcal/kg)	2,950	3,000	3,050
Ether extract (%)	4.294	5.799	5.581
Calcium (%)	1.027	0.88	0.831
Available phosphorus (%)	0.491	0.44	0.411
Chloride (%)	0.23	0.23	0.23
Sodium (%)	0.167	0.162	0.158
Methionine (%)2)	0.643(0.613)	0.569(0.543)	0.506(0.481)
Lysine (%)	1.478(1.34)	1.256(1.12)	1.104(0.984)
Arginine (%)	1.632(1.50)	1.512(1.38)	1.347(1.13)
Methionine+cystine (%)	1.083(0.987)	0.924(0.841)	0.835(0.759)
Threonine (%)	1.004(0.895)	0.856(0.755)	0.771(0.678)
Tryptophan (%)	0.298(0.254)	0.278(0.237)	0.246(0.210)

1) Supplied per kilogram of diet: retinol, 9,000 IU; alpha tochopherol acetate, 36 IU; cholecaciferol, 2,000 IU; cyanocobalamin, 0.015 mg; riboflavin, 6.6 mg; calcium pantothenate, 9.8 mg; niacin, 30 mg; choline chloride, 625 mg; biotin, 0.1 mg; thiamine, 1.75 mg; pyridoxine, 3 mg; folic acid, 1 mg; menadione, 2 mg; antioxidant (ethoxy queen), 100 mg; manganese, 248 mg; zinc, 211 mg; copper, 25 mg; iron, 125 mg; iodine, 2.5 mg; selenium, 0.5 mg .

2) Amino acid values represent both the total amino acid value and standardised ileal digestibility (SID) of amino acid value (in parentheses).

**Table 2:** The effects of dietary supplementation with glutamine (Gln) and arginine (Arg) on feed intake (g/bird/d), weight gain (g/bird/d), FCR, and mortality ratio in broiler reared under cold environmental temperature (7 to 42 days of age)

Arginine	Glutamine (0%)			Glutamine (0.5%)			Glutamine (1%)			SEM	p value		
			
	100%	130%	160%	100%	130%	160%	100%	130%	160%		Gln	Arg	Gln×Arg
Feed intake													
Starter1)	23.98	24.17	23.19	23.76	51.23	23.80	24.10	24.15	24.13	0.54	0.64	0.78	0.82
Grower	69.84	60.49	68.51	70.81	71.00	69.62	65.05	67.74	66.95	2.47	0.08	0.50	0.12
Finisher	140.10	145.30	144.40	150.00	163.30	165.10	143.30	135.80	136.60	10.75	0.056	0.87	0.85
Total	77.98	76.60	78.70	81.54	85.96	86.18	77.49	75.90	75.86	3.93	0.034	0.92	0.91
Weight gain													
Starter	15.96	15.90	15.42	15.60	15.97	16.05	15.99	16.48	16.16	0.44	0.45	0.73	0.83
Grower	31.16^ab^	28.47^b^	31.00^ab^	33.01^ab^	34.18^ab^	36.62^a^	29.92^ab^	31.34^ab^	29.58^ab^	1.57	0.001	0.64	0.37
Finisher	52.99^b^	62.52^ab^	65.30^ab^	68.57^ab^	72.39^ab^	75.37^a^	63.62^ab^	60.93^ab^	64.43^ab^	4.3	0.004	0.18	0.64
Total	33.36^c^	35.63^bc^	37.24^bc^	39.06^ab^	40.84^ab^	42.68^a^	36.51^bc^	36.25^bc^	36.71^bc^	1.66	0.0007	0.18	0.81
FCR													
Starter	1.50	1.51	1.50	1.50	1.46	1.48	1.49	1.46	1.48	0.03	0.53	0.70	0.92
Grower	2.25	2.17	2.21	2.25	2.18	1.98	2.18	2.15	2.27	0.101	0.61	0.66	0.40
Finisher	2.65	2.30	2.29	2.17	2.26	2. 17	2.27	2.21	2.11	0.11	0.031	0.16	0.35
Total	2.32	2.13	2.13	2.08	2.10	2.01	2.12	2.08	2.06	0.07	0.06	0.20	0.67
Ascites mortality (%)	46.60^a^	26.67^ab^	37.33^ab^	17.33^ab^	14.67^b^	22.67^ab^	18.67^ab^	32.00^ab^	17.33^ab^	7.63	0.003	0.85	0.12
RV/TV ratio	0.27.3^a^	0.15^b^	0.22^ab^	0.22^ab^	0.25^ab^	0.25^ab^	0.22^ab^	0.23^ab^	0.25^ab^	0.022	0.39	0.32	0.016

SEM, standard error of the mean; FCR, feed conversion ratio; RV, right ventricle; TV, total ventricular.

1) Starter (1–10 d), grower (11–24 d), finisher (25–42 d), total (1–42 d).

a,b Means in each row with no common superscript differ significantly (p<0.05).

**Table 3:** The main effects of dietary supplementation with glutamine (Gln) and arginine (Arg) on feed intake (g/bird/d), weight gain (g/bird/d), FCR, mortality and RV/TV ratio in broiler reared under cold environmental temperature (7 to 42 days of age)

Items	Glutamine levels			SEM	p value	Arginine levels			SEM	p value
	
	(0%)	(0.5%)	(1%)			100%	130%	160%		
Feed intake										
Starter1)	23.782	23.69	24.09	0.387	0.64	23.94	23.94	23.67	0.39	0.78
Grower	66.28	70.48	66.58	1.43	0.08	68.57	66.41	68.36	1.43	0.50
Finisher	143.3	159.52	138.59	6.21	0.055	144.5	148.17	148.73	6.21	0.87
Total	77.79^b^	84.56^a^	76.42^b^	2.27	0.034	79	79.51	80.25	2.27	0.92
Weight gain										
Starter	15.76	15.87	16.21	0.25	0.45	15.85	16.12	15.88	0.25	0.73
Grower	30.21^b^	34.61^a^	30.28^b^	0.909	0.001	31.36	31.33	32.4	0.91	0.64
Finisher	60.27^b^	72.11^a^	62.99^b^	2.48	0.004	61.72	65.28	68.36	2.48	0.18
Total	35.41^b^	40.86^a^	36.49^b^	0.96	0.0007	36.31	37.58	38.88	0.96	0.18
FCR										
Starter	1.5	1.48	1.48	0.017	0.53	1.5	1.48	1.49	0.02	0.70
Grower	2.21	2.14	2.2	0.056	0.61	2.23	2.17	2.15	0.056	0.66
Finisher	2.41^a^	2.2^b^	2.19^b^	0. 062	0.031	2.36	2.25	2.19	0. 06	0.16
Total	2.2	2.07	2.09	0.041	0.06	2.18	2.11	2.07	0.04	0.20
Ascites mortality (%)	36.88^a^	18.22^b^	22.66^ab^	3.88	0.003	27.55	24.44	25.77	3.88	0.85
RV/TV	0.21	0.24	0.23	0.013	0.39	0.24	0.21	0.24	0.01	0.32

SEM, standard error of the mean; FCR, feed conversion ratio; RV, right ventricle; TV, total ventricular.

1) Starter (1–10 d), grower (11–24 d), finisher (25–42 d), total (1–42 d).

a,b Means in each row with no common superscript differ significantly (p<0.05).

**Table 4:** The effects of dietary supplementation with Glutamine (Gln) and Arginine (Arg) on length, proportional weight of small intestine, and proportional weight of visceral organs in broiler reared under cold environmental condition

Arginine	Glutamine (0%)			Glutamine (0.5%)			Glutamine (1%)			SEM	p value		
			
	100%	130%	160%	100%	130%	160%	100%	130%	160%		Gln	Arg	Gln×Arg
Small intestinal length (cm)	152.8	152.8	149.6	156.2	164.6	144.2	159	152	164	6.6	0.5	0.74	0.24
Small intestinal weight1)	3.5	3. 03	2.5	3.44	3.14	3.33	2.81	3.25	3..21	0.41	0.67	0.78	0.52
Organs weight1)													
Liver	3.47	2.69	2.82	2.64	3.04	2.2	2.5	3.17	2.62	0.29	0.31	0.20	0.13
Gizzard	1.94	1.72	1.75	1.84	1.74	1.79	1.74	1.85	1.47	0.15	0.63	0.45	0.66
Pancreas	0.26	0.22	0.61	0.25	0.24	0.21	0.57	0.29	0.21	0.17	0.59	0.72	0.36
Spleen	0.2	0.14	0.12	0.18	0.12	0.13	0.13	0.14	0.12	0.021	0.45	0.06	0.38

SEM, standard error of the mean.

1) Proportional weight = weight segment or organ/final live body weight.

**Table 5:** The effects of dietary supplementation with glutamine (Gln) and arginine (Arg) on villus height, villus width, crypt depth, villus height/crypt depth ratio and goblet cell number (GCN) in small intestine of broiler reared under cold environmental condition

Arginine	Glutamine (0%)			Glutamine (0.5%)			Glutamine (1%)			SEM	p value		
			
	100%	130%	160%	100%	130%	160%	100%	130%	160%		Gln	Arg	Gln×Arg
Villus height (μm)													
Duodenum	1,530.30^d^	1,564.60^d^	1,710^ab^	1,665.30^bc^	1,782.60^a^	1,510^d^	1,585.60^cd^	1,501^d^	1,659.60^bc^	18.7	0.0007	0.12	0.0001
Jejunum	1,220.60^d^	1,247.00^d^	1,298.60^c^	1,366.30^b^	1,420.30^a^	1,230^d^	1,269.30^cd^	1,224^d^	1,297.30^c^	15.8	0.0001	0.26	0.0001
Ileum	852.60	884.00	867.30	859.30	907.60	871.60	872.60	881.60	885.00	20.4	0.72	0.23	0.86
Villus with (μm)													
Duodenum	125.60	128.60	121.00	124.60	115.30	127.30	129.60	130.30	125.00	3.46	0.14	0.70	0.08
Jejunum	127.00	122.30	131.30	117.00	118.60	130.60	121.60	125.60	123.30	3.48	0.48	0.14	0.41
Ileum	136.00abc	128.00c	154.60^a^	125.30^c^	135.30^bc^	151.00^ab^	134.60^bc^	131.30c	132.30^bc^	3.81	0.11	0.0002	0.005
Crypt deth (μm)													
Duodenum	121.60^bc^	120.30^bc^	121.60^bc^	135.6^ab^	147.30^a^	103.00^d^	122.00^bc^	107.00^cd^	123.00^bc^	3.46	0.002	0.003	0.0001
Jejunum	124.60^bc^	121.60bcd	117.00cde	137.00^ab^	142.60^a^	102.60^e^	127.30abc	104.00^de^	127.60abc	20.46	0.037	0.0006	0.0001
Ileum	93.66	88.00	84.33	89.00	81.33	71.66	82.00	81.00	90.00	5.25	0.12	0.33	0.20
Villi height/crypt ratio													
Duodenum	12.58^bc^	13.00abc	14.01^ab^	12.28^bc^	12.10^c^	14.67^a^	13.01abc	14.07^ab^	13.51abc	0.37	0.27	0.0005	0.011
Jejunum	9.80^c^	10.25abc	11.09abc	9.97^bc^	9.96^bc^	12.03^a^	9.97^bc^	11.80^ab^	10.18^bc^	0.37	0.59	0.003	0.002
Ileum	9.11	10.06	10.45	9.71	11.42	12.17	10.76	10.94	9.87	0.72	0.14	0.20	0.27
Goblet cell number (mm^2^)													
Duodenum	881.30^de^	1,006.6^bc^	1,053.30abc	1,006.00^c^	1,086.30^ab^	1,120.00^a^	805.33^e^	1,120^a^	978.00^cd^	20.46	0.0001	0.0001	0.30
Jejunum	1,248.60^a^	1,236.3^a^	1,273.00^a^	1,212.50^ab^	1,060.30^b^	1,171.30^ab^	1,204.30^ab^	1,230.30^ab^	1,230.60^ab^	33.05	0.001	0.26	0.09
Ileum	1,430.00^a^	1,410.3^ab^	1,409.30^ab^	1,298.00^bc^	1,286.70^c^	1,317.30abc	1,326.70abc	1,314.30abc	1,392.0abc	24.6	0.0002	0.23	0.48

SEM, standard error of the mean.

a,b Means in each row with no common superscript differ significantly (p≤0.05).
